# The effect of incorporating inorganic materials into quaternized polyacrylic polymer on its mechanical strength and adsorption behaviour for ibuprofen removal

**DOI:** 10.1038/s41598-020-62153-1

**Published:** 2020-03-23

**Authors:** Guang Zhang, Shuangshuang Li, Chendong Shuang, Yunsong Mu, Aimin Li, Liang Tan

**Affiliations:** 1 State Key Laboratory of Pollution Control and Resources Reuse, School of the Environment, Nanjing University, Nanjing, 210023 P. R. China; 2 China School of Environment & Natural Resources, Renmin University of China, Beijing, 100872 P. R. China; 3 Quanzhou Institute for Environmental Protection Industry, Nanjing University, Quanzhou, 362008 P. R. China

**Keywords:** Environmental impact, Pollution remediation

## Abstract

Quaternized polyacrylic polymer has many applications in water treatment because of its ion exchange effects, but its further industrial applications are largely restricted because of its poor mechanical strength. In this work, a magnetic anion exchange resin with a polyacrylic matrix (MAP) was prepared by incorporation of Fe_3_O_4_ and subsequent modification with tetraethyl orthosilicate (TEOS) to improve the mechanical strength and adsorption performance. The incorporation of Fe_3_O_4_ significantly enhanced the mechanical strength of the polymer and improved the sphericity rate after ball milling of the polyacrylic resin from 80.1% to 97.2% as a result of hydrogen bonding between the -OH groups on Fe_3_O_4_ and the -NH- groups on the resin matrix. Further TEOS modification could effectively prevent Fe_3_O_4_ particles from dislodging from the resins. The adsorption performance was evaluated by using ibuprofen as a model compound. The adsorption kinetics showed that adsorption equilibrium was reached in 150 min. XPS analysis indicated that hydrogen bonding greatly contributed to the adsorption of ibuprofen onto the MAP. Adsorption isotherm analysis indicated that the adsorption was endothermic.

## Introduction

Pharmaceuticals and personal care products (PPCPs) cause serious environmental problems because of their widespread presence in water and their potential toxicity, persistence, and bioaccumulation^1–3^. The removal of PPCPs from water has become increasingly of interest because the discharge of PPCPs threatens both human health and environmental systems. Several techniques have been applied for the removal of PPCPs, including biological treatment, adsorption, advanced oxidation processes, coagulation, and membrane filtration^4–7^. Among these methods, adsorption is commonly employed because of its simple and cost-efficient procedure^8,9^. As the most commonly used adsorbent, activated carbon (AC) has a high removal efficiency for many contaminants in water treatment due to its large surface area and abundant pores^4^. Although multiple interactions exist between AC and contaminants, including van del Waals interactions, π-π interactions, hydrogen bonding and electrostatic bonding, hydrophilic contaminants are generally more difficult to remove from aqueous solution because of their higher affinity for water molecules. Therefore, AC must be chemically modified before it can be used for water-soluble dye removal^10^. Some charged and hydrophilic contaminants, such as ibuprofen, DEET and gemfibrozil, can generally not be efficiently removed by AC^11,12^. Moreover, the adsorption capacity of AC is frequently decreased by pore-blocking and competition by natural organic matter (NOM)^6,13,14^.

Quaternized polyacrylic polymers, known as strongly basic anion exchange resins, are extensively used for the removal of organic matter in water treatment. Reportedly, magnetic ion exchange resin (MIEX) can remove both hydrophobic and hydrophilic dissolved organic matter (DOM) in drinking water pretreatment^15^. It was also reported that polyacrylic ion exchange resin could effectively remove humic acid and reactive dyes^16,17^. Furthermore, negatively charged PPCPs, including clofibric acid, diclofenac, and ibuprofen, could be effectively removed by magnetic ion exchange resin, with better performance than that of mesoporous silica SBA-15, a metal-organic framework, and modified inorganic-organic pillared clays^18–21^. However, due to its poor mechanical strength, polyacrylic ion exchange resins are susceptible to disintegration, generating particulate matter when used in a completely mixed contactor^16,22^. Neale *et al*. previously reported that particles with diameters of 5–10 μm could be observed during MIEX resin treatment^23^. In addition, many studies have indicated that MIEX pretreated membranes could be fouled by small particulate matter^22^.

In this paper, a novel magnetic anion exchange resin with a polyacrylic matrix (MAP) and high mechanical strength was prepared via a two-part procedure involving the incorporation of Fe_3_O_4_ and subsequent modification with tetraethyl orthosilicate (TEOS). Ibuprofen was chosen as a model negatively charged pharmaceutical, and its adsorption behaviour was systematically investigated under different solution chemistry conditions. The adsorption mechanism was investigated via X-ray photoelectron spectroscopy (XPS).

## Materials and methods

### Materials

Quaternized polyacrylic polymer beads with particle diameters of 0.4~0.6 mm and a strongly basic anion exchange capacity of 3.8 mmol/g, known as porous anion exchange resin D213 (AER) in China, were provided by Jiangsu Jinkai Resin Chemical Co. Ltd. (China). Amberlite IRA-958, purchased from J&K Chemical Co. Ltd. (China), served as a conventional anion exchange resin in experiments for comparison. Ferric chloride hexahydrate (FeCl_3_·6H_2_O), ferrous chloride tetrahydrate (FeCl_2_·4H_2_O), aqueous ammonia (25–28 wt%), methanol, sodium hydroxide (NaOH), sodium sulfate (Na_2_SO_4_), sodium chloride (NaCl), and hydrochloric acid (HCl, 37–39%) were of analytical grade and purchased from Sinopharm Chemical Reagent Co. Ltd. (China). Tetraethyl orthosilicate (TEOS, AR, >99%) was produced by Aladdin Industrial Corporation (Southern California, USA). Ibuprofen (IBF, > 98%) was purchased from Tokyo Chemical Industry Development Co. Ltd. (Shanghai, China). Humic acid (HA, molecular weight >10000), tannic acid (TA, C_76_H_52_O_46_, molecular weight = 1700), and gallic acid (GA, C_7_H_6_O_5_, molecular weight = 170) were obtained from J&K Chemical Co. Ltd. (China).

### Synthesis of the magnetic anion exchange resin

As illustrated in Fig. S1, the magnetic anion exchange resin with a polyacrylic matrix (MAP) was synthesized via a two-step procedure involving incorporation of Fe_3_O_4_ and modification with TEOS. In the first step, coprecipitation of Fe^3+^ and Fe^2+^ onto the resin matrix occurred^24^. Specifically, 40.00 g of FeCl_3_·6H_2_O and 17.16 g of FeCl_2_·4H_2_O were dissolved in 200 mL of deionized water with 10 g of anion exchange resin (AER) in a flask under a N_2_ atmosphere at room temperature and 200 rpm. The AER beads were collected by filtration and transferred to a 500 mL three-necked flask after 60 min. Then, 70 mL of ammonia solution (25–28 wt%) was added. The mixture was mechanically stirred at 200 rpm under a N_2_ atmosphere at 343 K. After 120 min, the obtained beads (Fe_3_O_4_-AER) were separated and washed with deionized water. In the second step, the obtained Fe_3_O_4_-AER, 10 mL of tetraethyl orthosilicate (TEOS), 100 mL of methanol, and 10 mL of ammonia solution (25–28 wt%) were mixed in a 500 mL three-necked flask and mechanically stirred at 200 rpm and 323 K for 120 min. The mixture was then naturally cooled to room temperature. Finally, the MAP beads were separated and washed with deionized water until the washing water reached neutral pH and were then dried at 313 K.

### Characterization

The sphericity rate after ball milling of the resins was typically employed to represent the mechanical strength of resin products and was calculated from the mass fraction of spherical resin after 30 min of ball milling (SQM-0.5, Changsha Tencan Powder Technology Co. Ltd., China), and the weight of all of the spherical resin beads and resin fragments were measured after drying at 318 K for 24 h. Scanning electron microscopy (SEM, FEI Quanta 250 FEG, USA) with energy dispersive spectroscopy (EDS, FEI Quanta 250 FEG, USA) was used to observe the surface morphology and confirm the surface elements of the resins. Fourier transform infrared spectroscopy (FTIR, Nexus870, Nicolet, USA) was used to identify the functional groups and analyse the improved mechanical strength of the resins. The crystalline structure of the iron oxide species was studied by X-ray diffraction (XRD, XTRA/3KW, Switzerland). To understand the magnetic properties of the resins, vibrating sample magnetometry (VSM, PerkinElmer, USA) was employed to obtain the magnetization curves of the resins. BET surface area was measured using nitrogen adsorption–desorption experiments (ASAP 2010, Micromeritics, USA). X-ray photoelectron spectroscopy (XPS, PHI 5000 VersaProbe, ULVAC-PHI, Japan) was utilized not only to analyse the reasons for the improved mechanical strength of the resins but also to investigate the interactions between the resins and ibuprofen. Notably, the XPS spectra were calibrated on the basis of the C1s bands at 284.6 eV.

### Adsorption

Batch adsorption experiments were carried out to study the adsorption behaviour of ibuprofen on MAP. For the kinetics studies, 0.1 g of MAP was shaken with 200 mL of ibuprofen solution with different initial concentrations (0.05 mM, 0.10 mM, and 0.15 mM) at 293 K and 150 rpm. Samples were taken at different time intervals for measurement within 320 min. Adsorption isotherms were acquired at different temperatures (278 K, 298 K and 318 K) from 0.05 g of MAP in 100 mL of ibuprofen solution with an initial ibuprofen concentration of 0.15 mM. To investigate the effect of pH from 2.0 to 11.5 on adsorption, the pH of 100 mL of ibuprofen solution (0.15 mM) was adjusted with 0.1 M HCl and 0.1 M NaOH. Then, 0.05 g of MAP was added, and the mixture was shaken at 293 K. Inorganic salt ions and organic matter are abundant in water and wastewater. Therefore, to evaluate the application of resins in practical water treatment engineering, inorganic salt ions with different valence states and organic matter with different molecular weights were used to investigate their effects on adsorption in this paper. The effects of coexisting inorganic salt ions (NaCl and Na_2_SO_4_) and coexisting organic matter (HA, TA, and GA) with concentrations ranging from 0 to 50 mg/L on the adsorption of ibuprofen onto MAP were evaluated at 293 K with 0.05 g of MAP in 100 mL of ibuprofen solution (0.15 mM). In addition, samples were taken from all the above experiments except for the kinetic experiments after 24 h and then analysed to ensure that the adsorption process could reach equilibrium. Except for the investigation of pH effect on adsorption, the pH in other experiments was not controlled.

The amount of ibuprofen adsorbed onto resin was calculated according to the following equation:

1 $${\rm{Q}}={\rm{V}}({{\rm{C}}}_{0}-{{\rm{C}}}_{{\rm{t}}})/{\rm{W}}$$

where Q (mmol/g) is the amount of ibuprofen adsorbed onto resin, V (L) is the volume of ibuprofen solution and W (g) is the weight of the dry resin. C_0_ (mmol/L) and C_t_ (mmol/L) represent the initial concentration of solution and the concentration of solution at time t (min), respectively.

### Reusability test

The reusability of MAP resin was evaluated by a typical adsorption-regeneration cycle test. MAP (0.1 g) was shaken with 200 mL of ibuprofen solution (0.15 mM) at 293 K and 150 rpm for 24 h. After adsorption, the resins were regenerated by 10 mL of NaCl solution (5%, w/w) at 293 K and 150 rpm for 24 h. After that, the resins were separated from the solution by pouring the water under a magnetic field. The separated resin was then washed with 10 mL of deionized water to reduce the influence of residual chloride ions on subsequent adsorption during reuse, and a parallel experiment was carried out using 100 mL of deionized water to wash the regenerated resin for comparison. The ibuprofen concentration in the solution after each adsorption cycle was measured, and the adsorbed amount was calculated according to Eq. (1).

## Results and discussion

### Characterization

**Figure 1:**
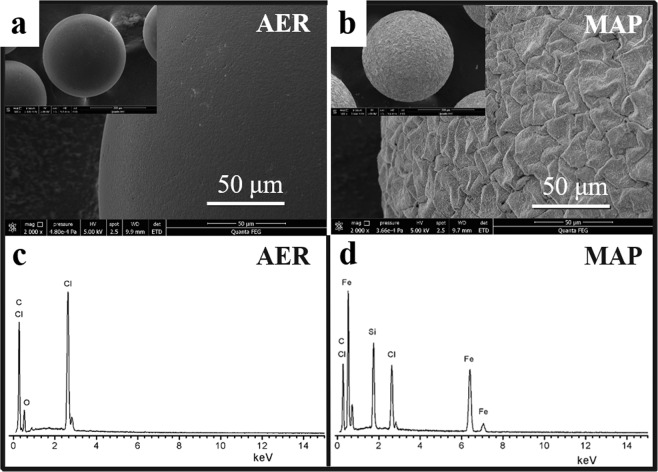
SEM images of the (**a**) AER and (**b**) MAP and EDS of the (**c**) AER and (**d**) MAP.

**Figure 2:**
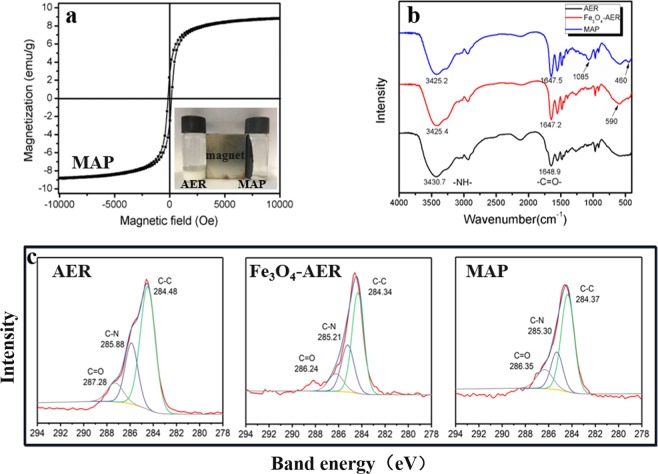
Characterization of the synthesized resins. (**a**) Magnetization curves of MAP. (**b**) FTIR spectra of the AER, Fe_3_O_4_-AER and MAP. (**c**) XPS spectra of the AER, Fe_3_O_4_-AER and MAP.

MAP was synthesized by incorporating Fe_3_O_4_ into the resins and then subsequent modification by TEOS. The colour of the obtained Fe_3_O_4_-AER and MAP was black, and the XRD results showed diffraction peaks such as (220), (311), (400), (422), (440), and (511), which confirmed the presence of Fe_3_O_4_ (Fig. S2). However, the water used for washing the Fe_3_O_4_-AER turned black, indicating that Fe_3_O_4_ particles were easily removed from the Fe_3_O_4_-AER matrix or surface during process because there was no resistance to the removal of Fe_3_O_4_ particles from the resin. As expected, TEOS could react with the hydroxyl group of Fe_3_O_4_ to form a dense layer; thus, the dense layer could protect the Fe_3_O_4_ stability from loss. This Fe_3_O_4_ loss could be greatly improved by the modification of the Fe_3_O_4_-AER resin with TEOS, as shown in Fig. S3. The surface of MAP is shown in Fig. 1a,b, where a scale-like and rough surface is visible. EDS analysis confirmed the existence of iron and silicon (Fig. 1c,d). The adsorption bands at 1085 cm^−1^ and 460 cm^−1^ in the FTIR spectra were attributed to the stretching vibrations of Si-O-Si and Si-O, respectively^25,26^. The adsorption band at 590 cm^−1^ of MAP, which was not in the FTIR spectra of AER, corresponded to the stretching vibration of Fe-O in Fe_3_O_4_ (Fig. 2b)^27^. These results indicated the successful preparation of MAP, which is illustrated in Fig. S1. Due to the existence of Fe_3_O_4_, the MAP possessed magnetic properties, and its specific saturation magnetization was 8.8 emu/g, which could be observed from the magnetization curves and resulted in effective separation under a magnetic field (Fig. 2a). The TGA results for AER, Fe_3_O_4_-AER, and MAP showed that the content of inorganic materials was calculated to be 17.4% (Fig. S4). In addition, the resin powder from grinding the beads had a uniform black colour, suggesting the presence of Fe_3_O_4_ both in the pores and on the surface of the resin. Notably, the porous structure of the polyacrylic anion exchange resin was difficult to analyse because the pores of these resins generally collapsed during drying, which was needed before determination of the pore structure and surface area. However, nitrogen adsorption-desorption experiments were also carried out. The BET surface areas were 0.23, 0.54 and 0.41 m^2^/g, and the average pore diameters were 13.9, 17.8 and 18.1 nm for the AER, Fe_3_O_4_-AER, and MAP, respectively.

**Figure 3:**
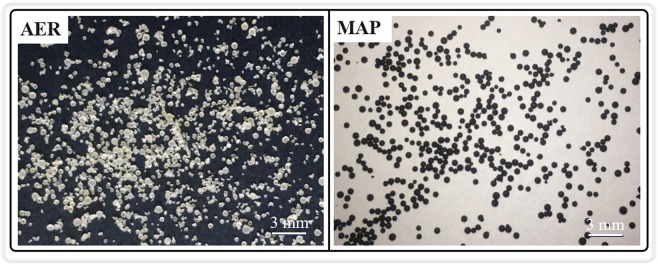
Photograph of the AER and MAP after ball milling.

Compared to commonly used polystyrenic resins, polyacrylic matrix resins have not been extensively employed because of their poor mechanical strength. In this work, the mechanical strength was determined from the rate of sphericity after ball milling. A portion of the AER was broken apart (Fig. 3), and the rate of sphericity of ion exchange resins after ball milling was 80.1%, 97.2%, and 97.9% for the AER, Fe_3_O_4_-AER, and MAP, respectively. This result indicated that the incorporation of Fe_3_O_4_ into the resin matrix could greatly improve its mechanical strength. The FTIR spectra of the resins showed that the absorption band of -NH- had shifted from 3430.7 cm^−1^ to 3425.4 cm^−1^ and the −C = O− band at 1648.9 cm^−1^ had not obviously shifted (Fig. 2b). This result could be explained by the formation of hydrogen bonds between the -OH groups on Fe_3_O_4_ and the -NH- groups on the resin matrix. In the C1s spectra from the XPS analysis, the C-N bond and C=O bond shifted from 285.88 eV and 287.28 eV to 285.21 eV and 286.24 eV, respectively, after the incorporation of Fe_3_O_4_. This might have been due to the influence of hydrogen bonding on the -NH- groups that connect to the C=O bond (Fig. 2c)^27–29^. A similar finding was recently reported by Yanagisawa *et al*., who found that the mechanical properties of polymeric materials could be improved by hydrogen bonding^30^.

### Adsorption kinetics

**Figure 4:**
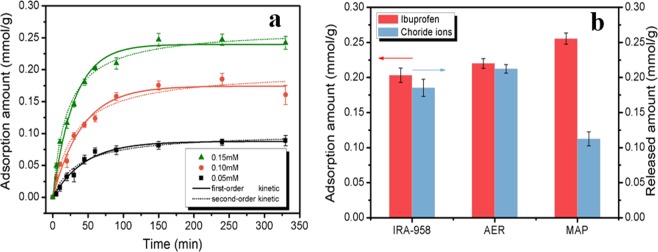
(**a**) Adsorption kinetics of ibuprofen onto the MAP with different initial ibuprofen concentrations of 0.05 mM, 0.10 mM, and 0.15 mM at 293 K and (**b**) the amount of chloride ions released from 0.1 g of AER and 0.1 g of MAP in 200 mL of ibuprofen solution with an ibuprofen concentration of 0.15 mM.

**Figure 5:**
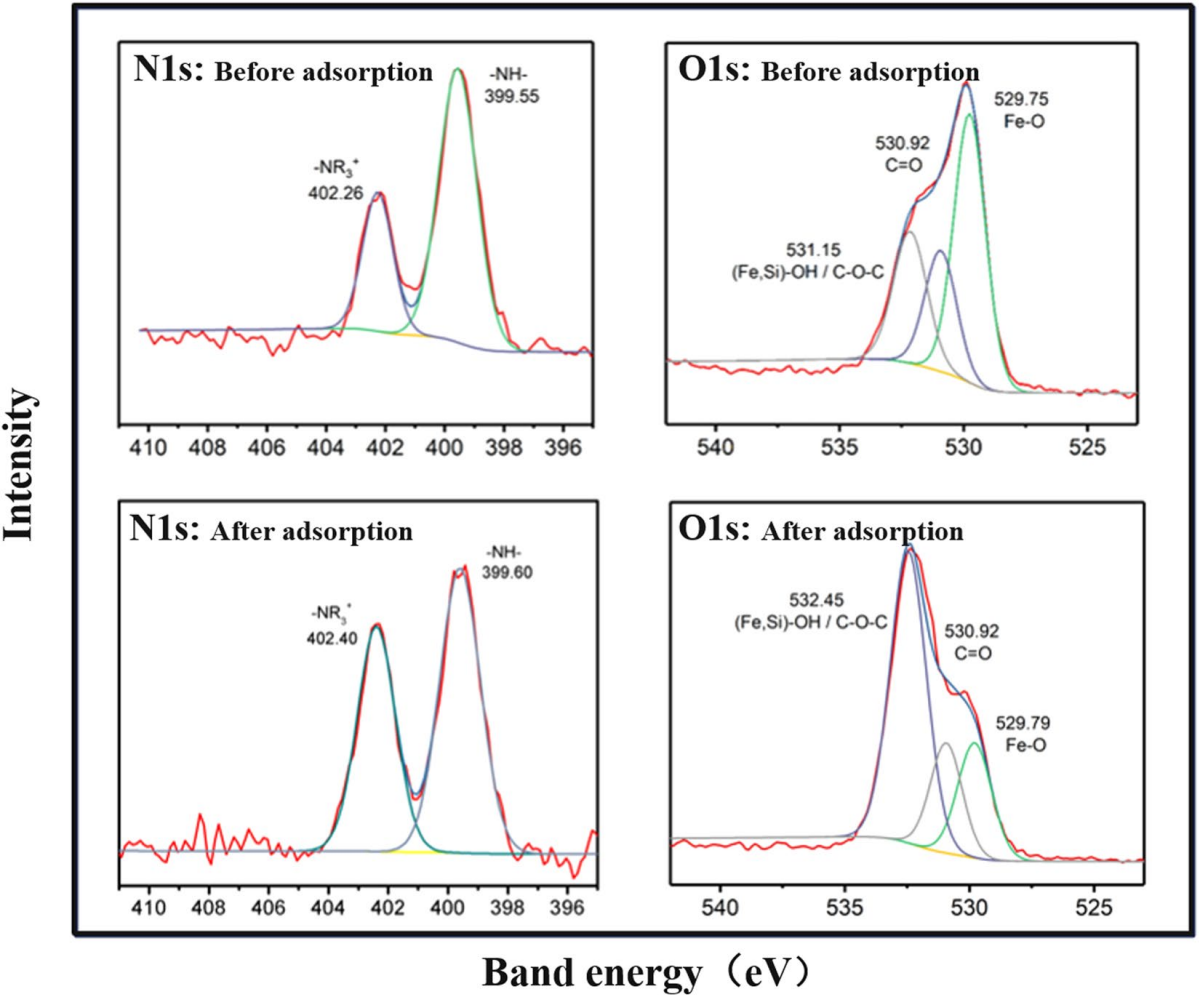
N1s and O1s XPS spectra of MAP before and after ibuprofen adsorption.

Figure 4a shows the adsorption kinetics of ibuprofen onto MAP at different initial concentrations of ibuprofen at 293 K. At the beginning of the 100 min period, adsorption rapidly increased in all of the MAP samples. Adsorption equilibrium was reached after 150 min. The amount of ibuprofen adsorbed onto the MAP at equilibrium increased as initial concentrations increased. To analyse the adsorption kinetics, the adsorption data were fitted by using pseudo-first-order kinetic and pseudo-second-order kinetic equations, both of which were the same as those in our previous work^16^. The fitting results are presented in Table 1, and both equations described the kinetics with correlation constants (*R*^2^) above 0.96. The fitting values of Q_e_ were consistent with the observed results. The adsorption of ibuprofen onto the MAP was caused by the electrostatic interactions between the negatively charged ibuprofen and the -NR_3_^+^ groups of the MAP. This result could be further confirmed by the zeta potentials shown in our previous results, in which we found that the quaternized polyacrylic resin is positively charged within a pH range of 2 to 12^16^. Therefore, the adsorption of ibuprofen onto the AER could be caused by electrostatic interactions. This was confirmed by the results that the chloride ions released was equimolar with adsorbed ibuprofen during adsorption. (Fig. 4b). The amount of ibuprofen adsorbed onto MAP was higher than that onto AER, while the amount of chloride ions released from MAP was approximately 50% less than that from AER. This result means that nonelectrostatic interactions contributed to ibuprofen adsorption onto MAP, which might be caused by inorganic materials. To further explore the interactions between MAP and ibuprofen, XPS was employed to analyse the MAP before and after the adsorption of ibuprofen. The result is shown in Fig. 5. The N1s XPS spectra were deconvoluted into two peaks at 399.55 eV and 402.26 eV, which were consistent with the presence of -NH- and -NR_3_^+^, respectively, in the resins. The peak corresponding to -NR_3_^+^ shifted from 402.26 eV to 402.40 eV after the adsorption of ibuprofen, possibly because of the electrostatic interactions between ibuprofen and -NR_3_^+^ ^31,32^. For the O1s spectra, three peaks at 531.15 eV, 530.92 eV, and 529.75 eV were assigned to the adsorbed oxygen or surface hydroxyl species ((Fe, Si)-OH/C-O-C), C=O bonds, and Fe-O bonds, respectively^27,33^. After the adsorption of ibuprofen, we found that the peak corresponding to (Fe, Si)-OH/C-O-C had shifted, which was related to the formation of hydrogen bonds between the -OH groups of inorganic materials on the MAP and the -COOH groups of ibuprofen. This finding was consistent with other reports, in which hydrogen bonding was considered the interaction between PPCPs and adsorbents^34–38^.

**Table 1:** Kinetic constants of ibuprofen adsorption onto MAP at 293 K.

MAP	First-order kinetic model			Second-order kinetic model			
	k_1_/10^−2^ (min^−1^)	Q_e_ (mmol/g)	*R*^2^	k_2_/10^−2^ (g/mmol min)	Q_e_ (mmol/g)	h_0_/10^−2^ (mmol/g min)	*R*^2^
0.05	2.21	0.09	0.9832	28.11	0.10	0.29	0.9823
0.10	2.43	0.185	0.9742	16.39	0.20	0.65	0.9795
0.15	3.28	0.24	0.9839	18.65	0.26	1.30	0.9699

### Adsorption isotherms

**Figure 6:**
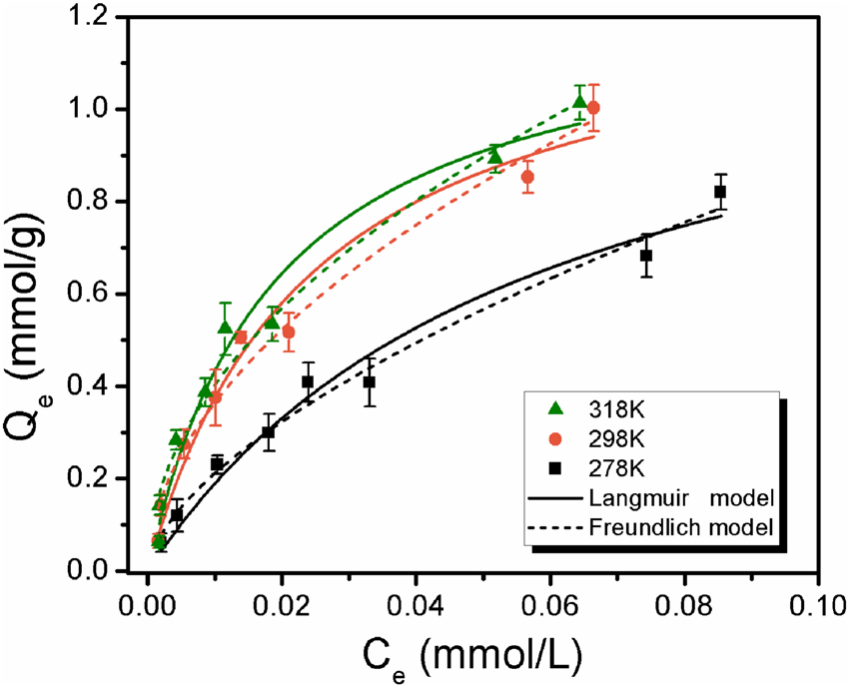
Adsorption isotherms of ibuprofen onto MAP under different temperatures of 278 K, 298 K, and 318 K with 0.05 g of MAP in 100 mL of ibuprofen solution.

The adsorption isotherms of ibuprofen onto MAP were investigated at temperatures of 278 K, 298 K, and 318 K (Fig. 6). The amount adsorbed onto MAP increased with increasing temperature, indicating that the adsorption of ibuprofen onto MAP was endothermic. The experimental data were fitted by the Langmuir model and the Freundlich model with correlation constants (*R*^2^) higher than 0.97, and the fitting results are displayed in Table 2. The Langmuir model and the Freundlich model were based on the following equations:

2 $${\rm{Langmuir}}\,{\rm{model}}:{{\rm{Q}}}_{{\rm{e}}}=\frac{{{\rm{Q}}}_{{\rm{m}}}{{\rm{K}}}_{{\rm{L}}}{{\rm{C}}}_{{\rm{e}}}}{1+{{\rm{K}}}_{{\rm{L}}}{{\rm{C}}}_{{\rm{e}}}}$$

3 $${\rm{Freundlich}}\,{\rm{model}}:{{\rm{Q}}}_{{\rm{e}}}={{\rm{K}}}_{{\rm{F}}}{{\rm{C}}}_{{\rm{e}}}^{1/{\rm{n}}}$$

where Q_e_ is the amount of ibuprofen adsorbed at equilibrium. In the Langmuir model, Q_m_ and K_L_ were the maximum adsorption capacity and the affinity of binding sites, respectively. From the fitting results, we observed that the affinity of binding sites increased with increasing temperature. In the Freundlich model, n and K_F_ represent the intensity and the Freundlich model constants, respectively. It was found that n increased as the initial temperature increased, and all the values of 1/n were less than 1, indicating that the adsorption of ibuprofen onto MAP was favourable according to Ammendola *et al*.^39^.

**Table 2:** Isotherm parameters from the Langmuir and Freundlich equations for ibuprofen adsorption onto MAP.

		Langmuir model			Freundlich model		
		K_L_ (L/mmol)	Q_m_ (mmol/g)	*R*^2^	n	K_F_ (L/g)	*R*^2^
MAP	278 K	17.29	1.29	0.9721	1.64	3.53	0.9840
	298 K	41.38	1.28	0.9721	1.92	3.99	0.9780
	318 K	53.08	1.25	0.9724	2.02	3.95	0.9702

### Effect of pH

**Figure 7:**
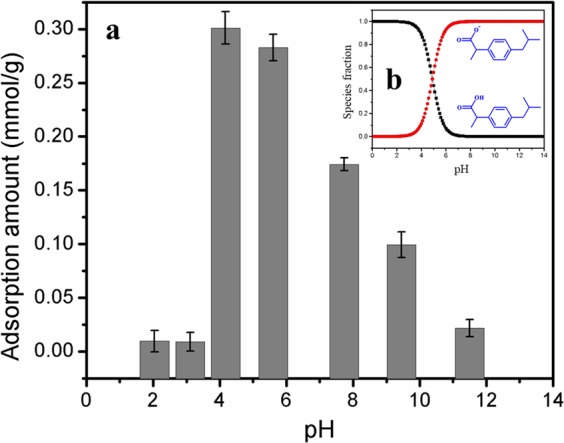
(**a**) Effect of solution pH on ibuprofen adsorption at 293 K with an initial ibuprofen concentration of 0.15 mM with 0.05 g of MAP in 100 mL of ibuprofen solution and (**b**) ibuprofen molecular/ion distribution at various pH values at 293 K.

The solution pH was an important factor that could exert a significant influence on the adsorption of ibuprofen onto MAP. As shown in Fig. 7, the maximum ibuprofen adsorption capacity of MAP was reached when the pH was 4.17. However, when the pH was lower than 4.17, the amount of ibuprofen adsorbed by the MAP greatly decreased because more ibuprofen was in the molecular form, which decreased the contribution of electrostatic interactions between anionic ibuprofen and -NR_3_^+^ in the resins. With the gradual increase in pH from 4.17, the negatively charged ionized ibuprofen species gradually became the dominant species due to the dissociation of the functional groups of ibuprofen, which contributed to the formation of electrostatic interactions. However, the adsorbed amount gradually decreased as the pH increased. According to the data calculations, the concentration of hydroxyl ions increased from 0.01 times to 21.1 times that of ibuprofen with the increase in pH from 8.0 to 11.5, which seriously decreased the adsorption of ibuprofen onto MAP due to the competition between the increasing amount of hydroxyl ions and the anionic ibuprofen species. This explanation of the phenomenon was consistent with previous work^40^. However, this effect of pH on adsorption was not consistent with the AC adsorption previously reported by Dubey *et al*., in which the amount of ibuprofen adsorbed by mesoporous honeycomb-structured AC gradually decreased with the gradual increase in pH from 2 to 8. This was attributed to the electrostatic repulsion generated between the negative charge on the AC surface and the negatively charged ibuprofen^41^.

### Effect of coexisting inorganic salt ions

**Figure 8:**
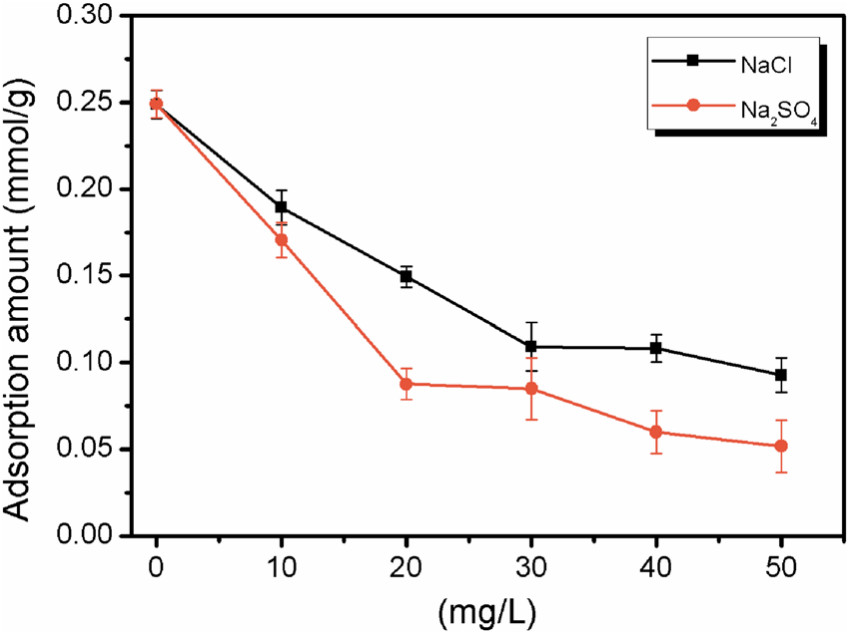
Effect of NaCl and Na_2_SO_4_ on ibuprofen adsorption onto MAP at 293 K with an initial ibuprofen concentration of 0.15 mM for 0.05 g of MAP shaken with 100 mL of ibuprofen solution.

The presence of inorganic salt ions in water or wastewater could influence adsorption behaviour via phenomena such as competitive adsorption and salting-out, which could respectively decrease and increase the adsorbed amount^40,42^. Therefore, to evaluate the effect of coexisting inorganic salt ions on ibuprofen adsorption onto resins in practical water treatment applications, chloride ions and sulfate ions were selected to represent monovalent and bivalent inorganic salt ions, respectively, in this paper. In this study, the adsorption of ibuprofen onto MAP at different concentrations of NaCl and Na_2_SO_4_ was used to further evaluate the adsorption behaviour (Fig. 8). As the concentration of NaCl and Na_2_SO_4_ increased from 0 mg/L to 50 mg/L, the amount of adsorbed MAP obviously decreased by 64% and 76%, respectively. This phenomenon could be interpreted as competition of the chloride ions and sulfate ions with the negatively charged ibuprofen species for the active ion exchange sites, and the sulfate ions were more negatively charged and displayed stronger inhibition than the chloride ions.

### Effect of coexisting organic matter

**Figure 9:**
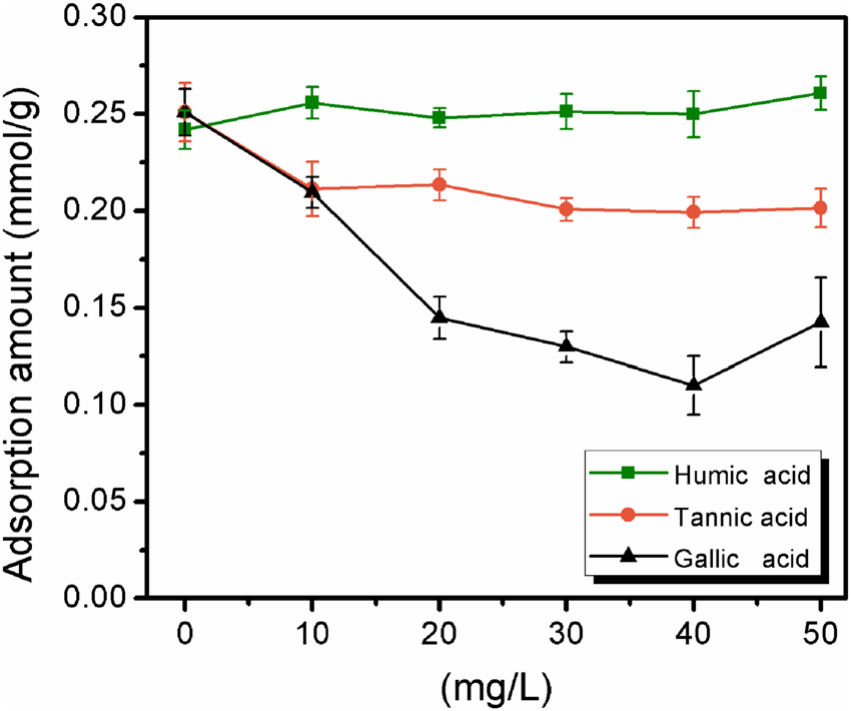
Effects of humic acid, tannic acid, and gallic acid on ibuprofen adsorption onto MAP at 293 K with an initial ibuprofen concentration of 0.15 mM for 0.05 g of MAP shaken with 100 mL of ibuprofen solution.

Although magnetic ion exchange resin treatment could effectively remove organic matter that commonly exists in water bodies, the treatment efficiency of the resins was significantly decreased after several cycles of regeneration, likely because of resin fouling by organic matter^43^. To evaluate the effect of organic matter on the removal of ibuprofen by MAP, organic matter species with different molecular weights, including HA, TA, and GA, were used as models to investigate their effect on adsorption in this work. Figure 9 shows that the amount of adsorbed MAP was not significantly influenced by the existence of HA, whereas as the concentration of TA and GA increased from 0 mg/L to 50 mg/L, the amount of adsorbed MAP decreased by 20% and 48%, respectively. These results might mean that TA and GA are more likely to diffuse into the pores of the MAP than HA due to their lower molecular weight than the other species. Another possibility was that TA and GA in solution were negatively charged when the pH was in the range of 4.5 to 6.0, allowing them to be adsorbed via electrostatic interactions, therefore resulting in competition^44^. It was also possible that the pore-blocking effect caused by TA and GA prevented ibuprofen molecules from diffusing into the resin pores^45^.

### Reusability

**Figure 10:**
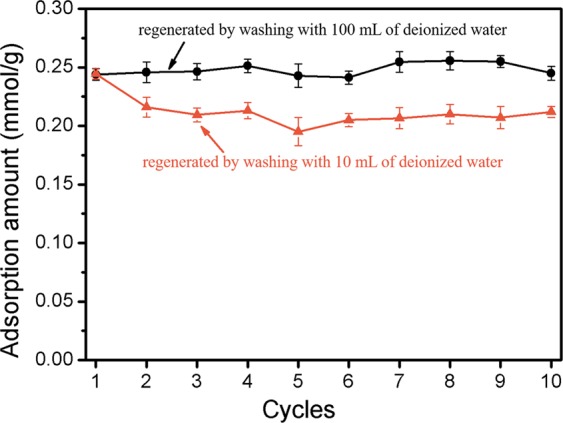
Adsorption-regeneration of MAP by 10 mL of NaCl solution (5%, w/w) and washing with 10 mL and 100 mL of deionized water.

The reusability of MAP for the adsorption of ibuprofen was evaluated by repeating the adsorption–desorption cycle 10 times. As shown in Fig. 10, the amount of ibuprofen adsorbed onto MAP was stable at 0.25 ± 0.02 mmol/g when the regenerated resin was washed with 100 mL of deionized water. This amount was comparable to the adsorption capacity of the fresh resin (0.25 mmol/g). However, this performance was questionable due to the large amount of waste produced, which was roughly equal to the volume of treated water. When the regenerated resin was washed with 10 mL of deionized water, the amount of ibuprofen adsorbed onto MAP (0.20 mmol/g) decreased by approximately 20% in the first 5 adsorption-regeneration cycles. However, the reusability stabilized at an adsorbed amount of 0.19 ± 0.02 mmol/g, suggesting that MAP is a feasible, reusable material for ibuprofen removal from water.

## Conclusions

In this work, a magnetic anion exchange resin with a polyacrylic matrix (MAP) was successfully prepared and characterized. The kinetics and isotherms of ibuprofen adsorption onto MAP were further investigated. The effects of solution pH, coexisting inorganic salt ions, and coexisting organic matter on ibuprofen adsorption were systematically evaluated. The main conclusions are summarized as follows:

1. The incorporation of Fe_3_O_4_ into polyacrylic AER resin could significantly improve the resin’s mechanical strength, as displayed by the 17.1% increase in the sphericity rate after ball milling. This could be attributed to the formation of hydrogen bonds between the -OH groups on Fe_3_O_4_ and the -NH- groups on the resin matrix.
2. While electrostatic interactions played an important role in MAP adsorption, hydrogen bonding between the -OH groups of the inorganic materials on MAP and the -COOH groups of ibuprofen also greatly contributed to the interactions.
3. Adsorption equilibrium was reached in 150 min. The adsorption isotherms of ibuprofen onto MAP were well described by both the Langmuir model and the Freundlich model, with correlation constants (R^2^) higher than 0.97. Adsorption is an endothermic process.
4. The amount of adsorption onto MAP decreased with increasing concentrations of coexisting salts NaCl and Na_2_SO_4_. Lower molecular weight organic matter had a significant influence on adsorption, with a 20% and 48% decrease in the presence of TA and GA (50 mg/L), respectively, whereas adsorption was not influenced by HA. The reusability performance showed that MAP could be reused at least 10 times.

## Supplementary information

Supplementary Information.

## References

[1] Bu Q, Wang B, Huang J, Deng S, Yu G (2013). Pharmaceuticals and personal care products in the aquatic environment in China: a review. J. Hazard. Mater..

[2] Grabicova K, Grabic R, Fedorova G, Fick J (2017). Bioaccumulation of psychoactive pharmaceuticals in fish in an effluent dominated stream. Water Res..

[3] Ebele AJ, Abdallah MA, Harrad S (2017). Pharmaceuticals and personal care products (PPCPs) in the freshwater aquatic environment. Emerg. Contam..

[4] Wang J, Wang S (2016). Removal of pharmaceuticals and personal care products (PPCPs) from wastewater: A review. J. Environ. Manag..

[5] Reungoat J, Escher BI, Macova M, Keller J (2011). Biofiltration of wastewater treatment plant effluent: effective removal of pharmaceuticals and personal care products and reduction of toxicity. Water Res..

[6] Kim SD, Cho J, Kim IS, Vanderford BJ, Snyder SA (2007). Occurrence and removal of pharmaceuticals and endocrine disruptors in South Korean surface, drinking, and waste waters. Water Res..

[7] Westerhoff P, Yoon Y, Snyder S, Wert E (2005). Fate of endocrine-disruptor, pharmaceutical, and personal care product chemicals during simulated drinking water treatment processes. Environ. Sci. Technol..

[8] Tan L (2018). Effect of pore structure on the removal of clofibric acid by magnetic anion exchange resin. Chemosphere..

[9] Hasan Z, Choi EJ, Jhung SH (2013). Adsorption of naproxen and clofibric acid over a metal–organic framework MIL-101 functionalized with acidic and basic groups. Chem. Eng. J..

[10] Gergo M, Frank PV, Font J, Fortuny A, Fabregat A (2012). Towards advanced aqueous dye removal processes: A short review on the versatile role of activated carbon. J. Environ. Manag..

[11] Bui TX, Choi H (2009). Adsorptive removal of selected pharmaceuticals by mesoporous silica SBA-15. J. Hazard. Mater..

[12] Wang Y (2016). Multi-walled carbon nanotubes with selected properties for dynamic filtration of pharmaceuticals and personal care products. Water Res..

[13] Mailler R, Gasperi J, Coquet Y (2015). Study of a large scale powdered activated carbon pilot: Removals of a wide range of emerging and priority micropollutants from wastewater treatment plant effluents. Water Res..

[14] Iovino P, Canzano S, Capasso S, Erto A, Musmarra D (2015). A modeling analysis for the assessment of ibuprofen adsorption mechanism onto activated carbons. Chem. Eng. J..

[15] Nguyen TV, Zhang R, Vigneswaran S (2017). Removal of organic matter from effluents by Magnetic Ion Exchange (MIEX®). Desalination..

[16] Shuang C (2012). Quaternized magnetic microspheres for the efficient removal of reactive dyes. Water Res..

[17] Shuang C, Wang M, Zhou Q, Zhou W, Li A (2013). Enhanced adsorption and antifouling performance of anion-exchange resin by the effect of incorporated Fe_3_O_4_ for removing humic acid. Water Res..

[18] Jiang M, Yang W, Zhang Z, Yang Z, Wang Y (2015). Adsorption of three pharmaceuticals on two magnetic ion-exchange resins. J. Environ. Sci..

[19] Lu X, Shao Y, Gao N, Chen J, Zhang Y (2016). Adsorption and removal of clofibric acid and diclofenac from water with MIEX resin. Chemosphere..

[20] Ma Y (2014). A bifunctional adsorbent with high surface area and cation exchange property for synergistic removal of tetracycline and Cu^2+^. Chem. Eng. J..

[21] Zhou Q (2014). Reusable magnetic microspheres for efficient removal of atrazine in aqueous media. Chem. Eng. J..

[22] Neale PA, Schäfer AI (2009). Magnetic ion exchange: is there potential for international development?. Desalination..

[23] Neale PA, Mastrup M, Borgmann T, Schäfer AI (2012). Sorption of micropollutant estrone to a water treatment ion exchange resin. J. Environ. Monit..

[24] Paulina M, Jakub M, Tiina L, Tomasz S, Czesław K (2019). Toward highly effective and easily separable halloysite-containing adsorbents: The effect of iron oxide particles impregnation and new insight into As(V) removal mechanisms. Sep. and Purif. Technol..

[25] Jin S, Park BC, Ham WS, Pan L, Kim YK (2007). Effect of the magnetic core size of amino-functionalized Fe_3_O_4_-mesoporous SiO_2_ core-shell nanoparticles on the removal of heavy metal ions. Colloids and surf., A..

[26] Ma Y (2014). Preparation of a novel magnetic microporous adsorbent and its adsorption behavior of p-nitrophenol and chlorotetracycline. J. Hazard. Mater..

[27] Ding C, Cheng W, Sun Y, Wang X (2015). Novel fungus-Fe_3_O_4_ bio-nanocomposites as high performance adsorbents for the removal of radionuclides. J. Hazard. Mater..

[28] Ramezanzadeh B, Ghasemi E, Mahdavian M, Changizi E (2015). Characterization of covalently-grafted polyisocyanate chains onto graphene oxide for polyurethane composites with improved mechanical properties. Chem. Eng. J..

[29] Jiang T, Kuila T, Kim NH, Ku BC, Lee JH (2013). Enhanced mechanical properties of silanized silica nanoparticle attached graphene oxide/epoxy composites. Compos. Sci. Technol..

[30] Yanagisawa Y, Nan Y, Okuro K, Aida T (2018). Mechanically robust, readily repairable polymers via tailored noncovalent cross-linking. Science..

[31] Li Q (2017). Preparation of permanent magnetic resin crosslinking by diallyl itaconate and its adsorptive and anti-fouling behaviors for humic acid removal. Sci. Rep..

[32] Li Q (2017). Competition and enhancement effect in coremoval of atenolol and copper by an easily regenerative magnetic cation exchange resin. Chemosphere..

[33] Chen Z, Wang J, Pu Z, Zhao Y (2017). Synthesis of magnetic Fe_3_O_4_/CFA composites for the efficient removal of U(VI) from wastewater. Chem. Eng. J..

[34] Kamarudin NHN, Jalil AA, Triwahyono S, Sazegar MR (2015). Elucidation of acid strength effect on ibuprofen adsorption and release by aluminated mesoporous silica nanoparticles. RSC Adv..

[35] Song JY, Jhung SH (2017). Adsorption of pharmaceuticals and personal care products over metal-organic frameworks functionalized with hydroxyl groups: Quantitative analyses of H-bonding in adsorption. Chem. Eng. J..

[36] Rubasinghege G, Gurung R, Rijal H (2018). Abiotic degradation and environmental toxicity of ibuprofen: Roles of mineral particles and solar radiation. Water Res..

[37] Bhadra BN, Ahmed I, Kim S, Jhung SH (2016). Adsorptive removal of ibuprofen and diclofenac from water using metal-organic framework-derived porous carbon. Chem. Eng. J..

[38] Ahmed I, Jhung SH (2017). Applications of metal-organic frameworks in adsorption/separation processes via, hydrogen bonding interactions. Chem. Eng. J..

[39] Ammendola P, Raganati F, Chirone R (2017). CO_2_ adsorption on a fine activated carbon in a sound assisted fluidized bed: thermodynamics and kinetics. Chem. Eng. J..

[40] Wang J (2015). Effect of pore structure on adsorption behavior of ibuprofen by magnetic anion exchange resins. Microporous Mesoporous Mater..

[41] Dubey SP, Dwivedi AD, Sillanpää M, Gopal K (2010). Artemisia vulgaris-derived mesoporous honeycomb-shaped activated carbon for ibuprofen adsorption. Chem. Eng. J..

[42] Shuang C, Pan F, Zhou Q, Li A, Li P (2012). Magnetic polyacrylic anion exchange resin: preparation, characterization and adsorption behavior of humic acid. Ind. Eng. Chem. Res..

[43] Li H, Li A, Shuang C, Zhou Q, Li W (2014). Fouling of anion exchange resin by fluorescence analysis in advanced treatment of municipal wastewaters. Water Res..

[44] Wang J, Li A, Xu L, Zhou Y (2009). Adsorption of tannic and gallic acids on a new polymeric adsorbent and the effect of Cu(II) on their removal. J. Hazard. Mater..

[45] Zhu Z, Xie J, Zhang M, Zhou Y, Liu F (2016). Insight into the adsorption of PPCPs by porous adsorbents: Effect of the properties of adsorbents and adsorbates. Environ. Pollut..

