# Supplementary material for: The effect of incorporating inorganic materials into quaternized polyacrylic polymer on its mechanical strength and adsorption behaviour for ibuprofen removal
Source: Sci Rep. 2020 Mar 23;10:5188. doi: 10.1038/s41598-020-62153-1 (PMC7090011; doi:10.1038/s41598-020-62153-1)

**Supplementary Information for**

**The effect of incorporating inorganic materials into quaternized polyacylic polymer on its mechanical strength and adsorption behaviour for ibuprofen removal**

Guang Zhang^1^, Shuangshuang Li^1^, Chendong Shuang^1^*, Yunsong Mu^2, 3^**, Aimin Li^1^, Liang Tan^1^

1 State Key Laboratory of Pollution Control and Resources Reuse, School of the Environment, Nanjing University, Nanjing 210023, P.R. China

2 State Key Laboratory of Environmental Criteria and Risk Assessment, Chinese Research Academy of Environmental Sciences, Beijing 100012

3 China School of Environment & Natural Resources, Renmin University of China, 100872

* Tel.: +86-25-89681669. Fax: +86-25-89681669.

E-mail: [shuangchendong@nju.edu.cn](mailto:shuangchendong@nju.edu.cn). (Shuang, C.D.), muys@craes.org.cn(Mu, Y.S.)

* Tel.: +86-25-89681669, +86-10-84915312.

**Contents**

Figure S1 The procedure for the synthesis of MAP.

Figure S2 Characterization of the Fe_3_O_4_-AER by XRD analysis.

Figure S3 Photograph of the AER, Fe_3_O_4_-AER, and MAP during the washing process.

Figure S4 TGA curves of the AER, Fe_3_O_4_-AER, and MAP.

**Figure S1**


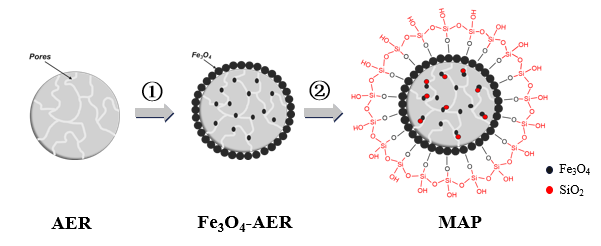


**Figure S2**


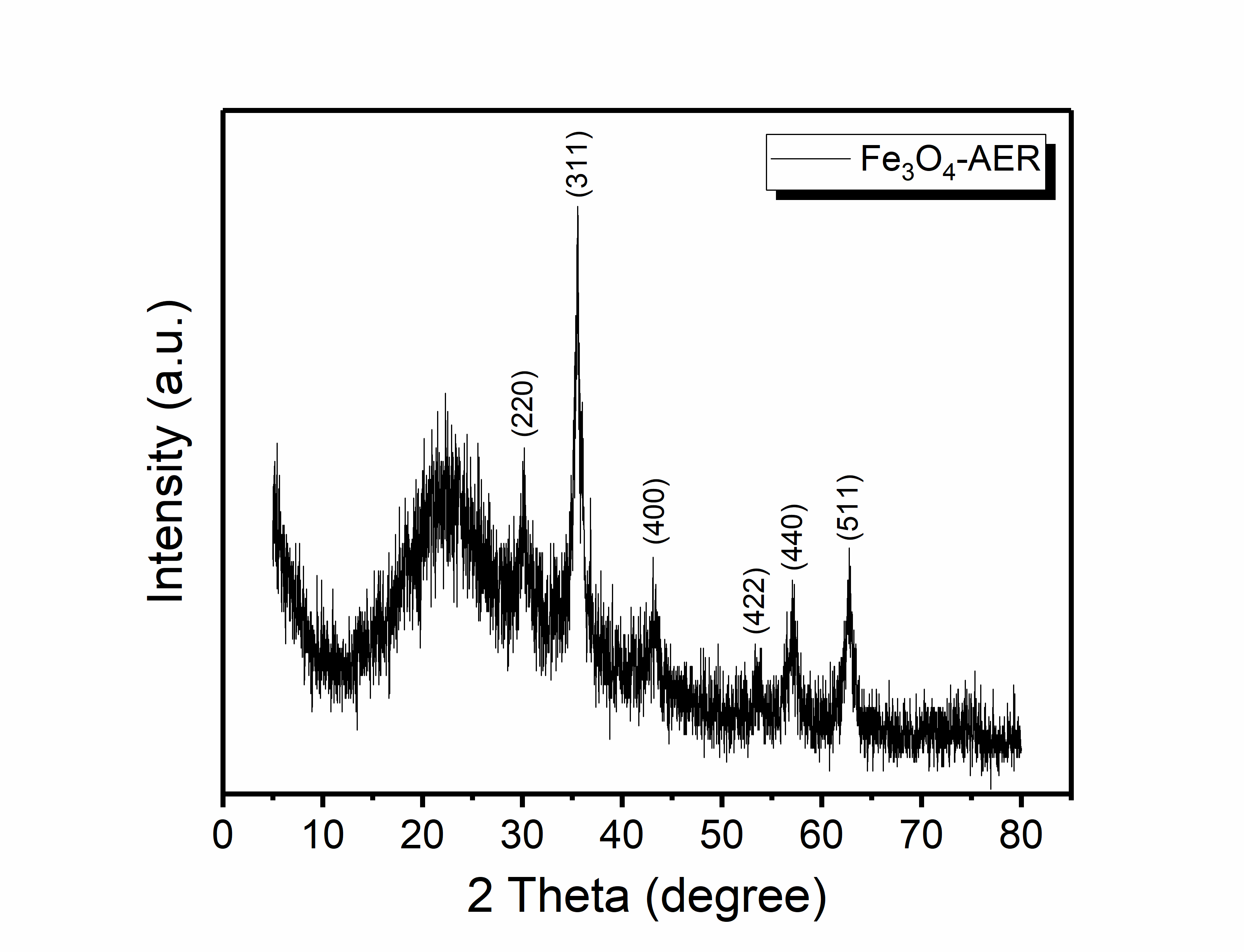


**Figure S3**


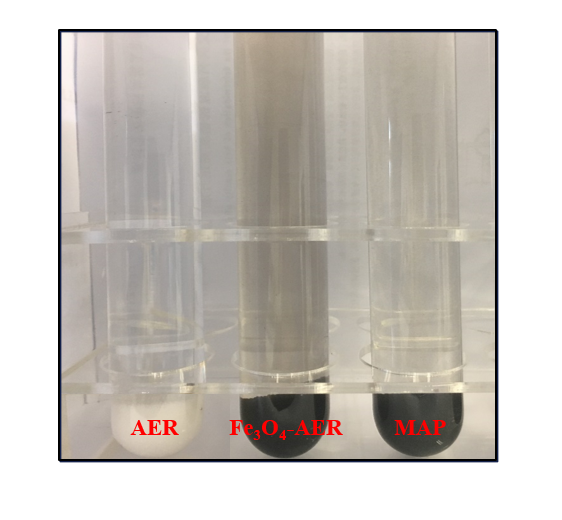


**Figure S4**


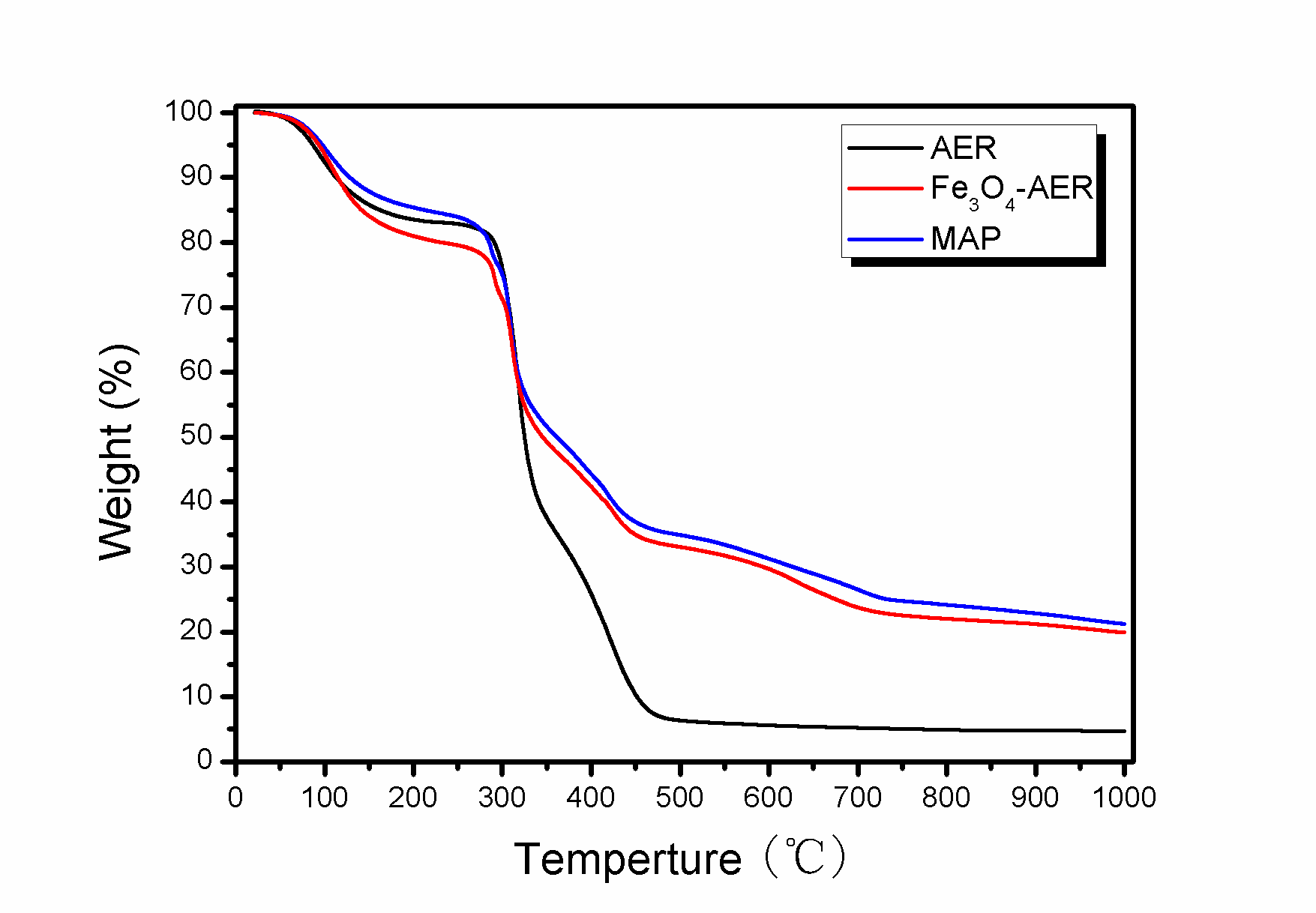

Supplement: Supplementary file 1 — Supplementary Information. [file 41598_2020_62153_MOESM1_ESM.docx]
